# Trends in CT use in an emergency department in Western Australia: 2015–2022

**DOI:** 10.1186/s13244-025-01993-9

**Published:** 2025-06-04

**Authors:** Fouziah Almouqati, Thi Ninh Ha, Sharmani Barnard, Ashu Gupta, Elizabeth Thomas, Tracey Bhar, Colleen Taylor, Delia Hendrie

**Affiliations:** 1 Curtin School of Population Health, Faculty of Health Sciences, Curtin University, Perth, Western Australia Australia; 2 Health Administration, College of Business Administration, King Saud University, Riyadh, Saudi Arabia; 3 Health Economics and Data Analytics, Curtin School of Population Health, Faculty of Health Sciences, Curtin University, Perth, Western Australia Australia; 4 Radiology Department, Fiona Stanley Hospital, Murdoch, Western Australia Australia; 5 Emergency Department, Fiona Stanley Hospital, Murdoch, Western Australia Australia

**Keywords:** Computed tomography, Emergency department, Imaging utilization, Overuse, Radiology

## Abstract

**Objectives:**

We examined trends in CT use within the emergency department (ED) and their association with trends in subsequent hospital admission.

**Methods:**

This retrospective study analyzed administrative data on episodes of adults aged 18+ years who presented to the ED of a tertiary hospital in Western Australia (WA) from March 2015 to December 2022. Adjusted annual rates of CT use and hospital admission, stratified by CT status, were estimated using multivariable regression models.

**Results:**

Between 2015 and 2022, while the number of ED episodes increased by 8%, the number of CT scans rose by 90%. The crude rate of scans per 1000 ED episodes rose from 111 [95% CI: 108, 113] to 195 [95% CI: 192, 199]. After adjusting for variations in patients’ characteristics, the rate increased from 118 [95% CI: 115, 121] to 173 [95% CI: 169, 176]. Admission rates were consistently higher for patients with CT but declined over time in both groups: from 47.6% [95% CI: 46.46, 48.75] to 42.01% [95% CI: 41.12, 42.9] for those with CT, and from 27.25% [95% CI: 26.86, 27.64] to 23.83% [95% CI: 23.47, 24.2] for those without. Compared to those without CT, the admission rate in those who underwent CT decreased by 2.17% [95% CI: 3.68, 0.66] over the period.

**Conclusions:**

CT use in the ED has continued to increase since 2015, coinciding with a greater decrease in admissions among patients who underwent CT. The appropriateness of this increase remains undetermined, warranting further investigation.

**Critical relevance statement:**

Given the ongoing efforts to optimize CT scan use, this study evaluates its current utilization in the emergency department and its usefulness in patient management, particularly in hospital admission.

**Key Points:**

- Examining CT use and usefulness is vital given ongoing optimization efforts.
- CT rates rose significantly, with a clear upward shift from 2020.
- This coincided with a greater drop in admission for CT patients than non-CT.

**Graphical Abstract:**

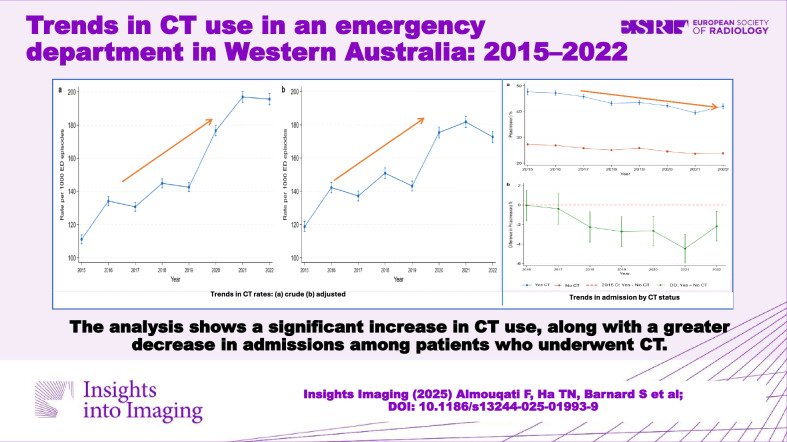

## Introduction

CT is the most widely used and fastest-growing advanced imaging technology, primarily driven by emergency departments (EDs) [1–4]. However, growing concerns suggest that part of this increase may reflect overuse (the provision of care that confers little to no benefit while being costly and potentially harmful) [5]. These concerns stem from the disproportionate rise in CT scans relative to ED visit volumes [6]. Furthermore, despite this rise, the diagnostic yield has remained stable, and approximately 27% of CT scans are being ordered outside clinical guidelines [7–9].

Frequent use of CT may pose significant risks. Although radiation from a single CT is minimal, repeated exposure can increase the risk of cancer [10]. Additionally, it may lead to overdiagnosis (the detection of asymptomatic conditions that would not cause harm), prompting further tests and treatments and potentially raising patient anxiety [11, 12]. Additionally, CT use increases healthcare expenditures, with some studies showing no improvement in outcomes [13–15].

During the past two decades, Australia has been actively promoting the use of clinically relevant imaging. In 2005, the Department of Health in Western Australia (WA) introduced Diagnostic Imaging Pathways, a set of evidence-based guidelines and an educational tool to help clinicians decide when imaging is needed and what type to use [16]. In 2015, the Choosing Wisely Australia campaign, modeled after the American Board of Internal Medicine’s 2012 initiative, was launched by the National Prescribing Service MedicineWise to reduce unnecessary medical procedures [17]. Specifically, regarding imaging use in the ED, the Royal Australian and New Zealand College of Radiologists recommends against performing CT scans for certain conditions unless indicated by pre-determined, validated clinical decision rules [18]. While the impact of this campaign has been mixed, some studies have reported improvements in imaging use [19–22]. A 15% reduction in the overuse of imaging for staging breast cancer was reported, and, in the ED, the use of head CT decreased significantly from 12.4 to 9.3%, while the diagnostic yield increased from 22.4 to 37.1% [21, 22].

Since the introduction of this campaign in 2015, studies on CT utilization in the ED have been limited [23, 24]. Recent evidence indicates a significant increase in CT use, though data is only available up to 2015 [23]. Furthermore, this evidence does not account for variations in physician experience, a known predictor of CT utilization [23, 25]. While utilization assessment provides a starting point in areas with ongoing policy efforts, it alone does not fully capture the value of CT. Though not ideal, one approach is to examine its impact on clinical management outcomes, such as hospital admission [6, 26]. This study primarily examined trends in CT use in an ED in WA from 2015 to 2022 and secondarily evaluated changes in admission rates among patients with and without CT scans over the same period.

## Materials and methods

### Study design and setting

This retrospective study was approved by an institutional review board with a waiver of individual consent. It analyzed de-identified, individual-level administrative data from adults aged 18+ who presented to the ED of a public tertiary hospital in WA. Reporting adhered to the Reporting of Studies Conducted Using Observational Routinely-Collected Health Data checklist.

### Data sources and processing

Data were extracted from two systems and organized into datasets:

1. ED Information System contains records from January 1, 2015, to December 31, 2022, covering basic clinicodemographics, visit specifics, clinician titles, and disposition status.
2. Radiology Information System includes CT records requested by the ED within the same timeframe, detailing the date, time, and scan code (classifying the type of scan).

The two datasets were merged chronologically using patient pseudonymous ID and event timestamp variables. CT records with matching IDs and timestamps falling between presentation and disposition were linked to the corresponding ED episode.

### Study population

The study population included all ED episodes for individuals aged 18+ from March 1, 2015, to December 31, 2022. January and February 2015 were excluded because they had substantially fewer ED episodes compared to subsequent months in that year and to the same months in subsequent years. Episodes marked as inpatient transfers or follow-ups were excluded as they had already commenced, and decisions on CT could be biased by prior treatments and tests not applicable to new cases. All exclusion criteria and the number of excluded records are listed in Fig. S1 (Supplementary Material).

### Outcomes

The primary outcome was the number of CT scans per 1000 ED episodes. For each ED episode, CT scans were counted by treating each scanned anatomical area as a single scan. Multiple records of the same area performed simultaneously were combined into a single scan to avoid overcounting and maintain consistency with earlier research [15]. We also analyzed the rates by anatomical area, identified using scan code. These included the head, neck, chest, spine, abdomen/pelvis, and others (extremities and face). The secondary outcome was the probability (expressed as a percentage) of an ED episode resulting in hospital admission. A case was considered admitted if either the disposition status indicated admission or an admission date was recorded.

### Covariates

The primary regressor was the year of presentation. We also adjusted for specific patient, clinician, and visit-level characteristics [23, 25, 27]. Patient characteristics included demographics such as age, sex, Indigenous status (excluded from reporting due to ethical considerations), and socioeconomic status, as well as clinical factors like triage (urgency of the patient’s need for care) and presenting symptoms (reason for the ED encounter). Socioeconomic status was identified using the Index of Relative Socioeconomic Advantage and Disadvantage, assigned based on patient postcode, with the 2016 and 2021 censuses providing state-scale quantiles for episodes from 2015–2018 and 2019–2022, respectively. Presenting symptoms were categorized into parent categories, following the methodology of a prior study [23]. Clinician titles were grouped into: senior physicians (e.g., consultants and registrars, authorized to order CT), junior doctors (e.g., residents and interns, not independently authorized to order CT), and other clinicians (e.g., physiotherapists). Visit characteristics included referral source, arrival mode, and day and time of shift. To assess changes in hospital admission based on CT status, a binary variable for CT use was added alongside all previous covariates. Claim type was excluded from analyses due to multicollinearity and imbalance, with 93% of episodes involving public coverage. Finally, for each variable, if fewer than 2000 records had missing data, these were grouped with “other”; otherwise, they were added as a separate category.

### Statistical analysis

Descriptive statistics were used to summarize annual population characteristics and CT scan counts from 2015 to 2022. To facilitate examination of changes over time, only 2015 and 2022 are presented in Table 1, with full annual data available in the Supplementary Material (Table S1). A multivariable negative binomial model accounting for overdispersion was used to examine the overall CT trend [28]. Additionally, six separate models analyzed CT per anatomical area. Post-estimation predictive margins, controlling for the variability in other covariates, were used to present the annual rates and confidence intervals (CIs) for overall CT and CT per anatomical area. To track the pace of change in the annual rate of CT per anatomical area, contrasts of margins by year were applied, subtracting the base year’s (2015) absolute rate from each subsequent year’s rate for each CT type.

**Table 1:** Number of CT scans and characteristics of the study population in 2015, 2022, and the overall study period

	2015	2022	Total
*N*	58,882 (10.6)	63,546 (11.5)	553,426 (100.0)
Overall CT	6541	12,428	85,376
Head	3500	5746	40,571
Neck	256	816	5199
Chest	483	1004	6889
Spine	633	1428	9381
Abdomen/pelvis	1280	2737	18,409
Other	389	697	4927
Episode characteristics			
CT use status			
No	53,229 (90.4)	53,864 (84.8)	484,447 (87.5)
Yes	5653 (9.6)	9682 (15.2)	68,979 (12.5)
Age group (years)			
18–34	18,793 (31.9)	17,042 (26.8)	161,785 (29.2)
35–54	16,616 (28.2)	17,369 (27.3)	154,613 (27.9)
55–74	13,048 (22.2)	15,136 (23.8)	130,744 (23.6)
75+	10,425 (17.7)	13,999 (22.0)	106,284 (19.2)
Median age (years)^a^	47 (31–67)	51 (33–72)	49 (32–69)
Sex			
F	29,787 (50.6)	32,379 (51.0)	284,420 (51.4)
M	29,095 (49.4)	31,167 (49.0)	269,006 (48.6)
Triage code			
Semi/non-urgent	25,919 (44.0)	19,780 (31.1)	213,155 (38.5)
Urgent	21,852 (37.1)	24,937 (39.2)	214,744 (38.8)
Resuscitation/emergency	11,111 (18.9)	18,829 (29.6)	125,527 (22.7)
Symptom group			
Other	11,814 (20.1)	11,668 (18.4)	109,112 (19.7)
Cardiovascular	1415 (2.4)	2102 (3.3)	16,247 (2.9)
Gastrointestinal	3301 (5.6)	3340 (5.3)	29,585 (5.3)
Injury	11,314 (19.2)	10,967 (17.3)	99,390 (18.0)
Neurological	5264 (8.9)	7334 (11.5)	54,851 (9.9)
Pain	19,196 (32.6)	19,761 (31.1)	178,165 (32.2)
Provisional diagnosis	1784 (3.0)	2476 (3.9)	20,587 (3.7)
Respiratory	3710 (6.3)	4694 (7.4)	35,474 (6.4)
Urology	1084 (1.8)	1204 (1.9)	10,015 (1.8)
Treating clinician			
Physician: Senior	17,709 (30.1)	16,101 (25.3)	182,309 (32.9)
Doctor: Junior	34,966 (59.4)	40,016 (63.0)	313,541 (56.7)
Other clinicians	6207 (10.5)	7429 (11.7)	57,576 (10.4)
Referral source^b^			
Self/relative	50,866 (86.4)	53,596 (84.3)	459,127 (83.0)
GP/OPD/hospital	5397 (9.2)	5877 (9.2)	59,686 (10.8)
Other	2499 (4.2)	3698 (5.8)	29,407 (5.3)
Arrival means			
Ambulance/police/flying	18,614 (31.6)	24,365 (38.3)	183,890 (33.2)
Other	40,268 (68.4)	39,181 (61.7)	369,536 (66.8)
Presentation day			
Weekday	41,529 (70.5)	44,727 (70.4)	391,485 (70.7)
Weekend	17,353 (29.5)	18,819 (29.6)	161,941 (29.3)
Presentation shift			
09:00–17:00	28,749 (48.8)	32,685 (51.4)	276,186 (49.9)
17:01–23:59	19,251 (32.7)	19,338 (30.4)	175,730 (31.8)
00:00–08:59	10,882 (18.5)	11,523 (18.1)	101,510 (18.3)
IRSAD^b^			
Least disadvantaged	27,846 (47.3)	23,346 (36.7)	234,976 (42.5)
Less	4845 (8.2)	9453 (14.9)	62,601 (11.3)
Moderate	14,416 (24.5)	16,551 (26.0)	140,296 (25.4)
Highly	6717 (11.4)	7362 (11.6)	63,138 (11.4)
Most disadvantaged	3046 (5.2)	4969 (7.8)	34,907 (6.3)
Admission status			
No	42,829 (72.7)	45,011 (70.8)	399,001 (72.1)
Yes	16,053 (27.3)	18,535 (29.2)	154,425 (27.9)

Unless otherwise noted, data are reported as *n* (%). Indigenous status is not reported due to ethics requirements; adjustments applied

*GP* general practitioner, *OPD* outpatient department, *IRSAD* Index of Relative Socioeconomic Advantage and Disadvantage

^a^ Reported as median age (IQR: 25th–75th)

^b^ Totals do not sum to 100% due to missing data

A multivariable logistic regression model was used to examine trends in hospital admission following an ED episode. Post-estimation predictive margins presented the annual probability of admission and CIs for those with and without CT. Contrasts of margins by year and CT status were used to analyze changes in the difference in admission rates between those with and without CT. This approach subtracted the baseline year’s admission rate difference between the two groups from differences in subsequent years, allowing assessment of significant changes in CT’s incremental impact on admission over time while controlling for unobserved factors, in line with prior research [29].

All models were specified with cluster-robust standard errors using patient IDs to account for repeat presentations. Analyses and visualizations were performed using Stata SE V.18 [30].

## Results

After applying the study population exclusion criteria, a total of 553,426 unique ED episodes remained, which included 85,376 CT scans (some episodes involving > 1 CT) (Table 1). The median patient age was 49 years: IQR: 32–69 years, with 51.4% of patients being female.

Between 2015 and 2022, the number of ED episodes increased by 8%, with steady growth until a consistent decline began in 2020 (Table S1, Supplementary Material). In contrast, the number of CT scans rose by 90%, showing consistent annual increases, except for a slight decrease in 2022 compared to 2021. Most patient characteristics displayed minimal changes over time; however, notable shifts were observed in age, triage, and the Index of Relative Socioeconomic Advantage and Disadvantage between 2015 and 2022. The median age rose from 47 years in 2015 to 51 years in 2022. By 2022, 29.6% of patients were categorized as resuscitation/emergency, and 7.8% resided in the most disadvantaged areas, representing 69 and 63% increases, respectively, compared with 2015. The percentage of patients admitted to the hospital also increased, from 27.3% in 2015 to 29.2% in 2022.

### Changes in CT rates

**Fig. 1:**
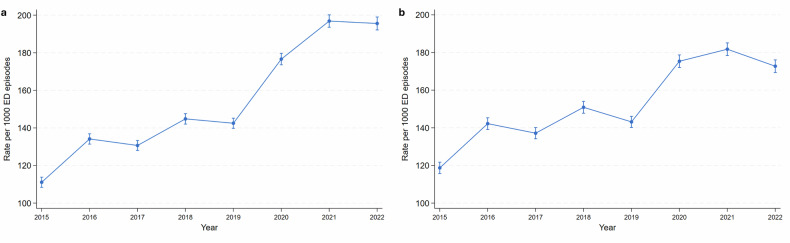
Trends in the rate of CT scanning. **a** The crude overall rate. **b** The adjusted overall rate

The crude rate of CT scans per 1000 ED episodes increased from 111 in 2015 [95% CI: 108, 113] to 195 in 2022 [95% CI: 192, 199], with an apparent upward deflection beginning in 2020 (Fig. 1a). After adjusting for patient, clinician and, visit factors (Table 1), the rate rose from 118 in 2015 [95% CI: 115, 121] to 173 in 2022 [95% CI: 169, 176], with the deflection in and following 2020 remaining evident (Fig. 1b). Further details on the regression for adjusted CT rates are shown in Table S2 (Supplementary Material).

**Fig. 2:**
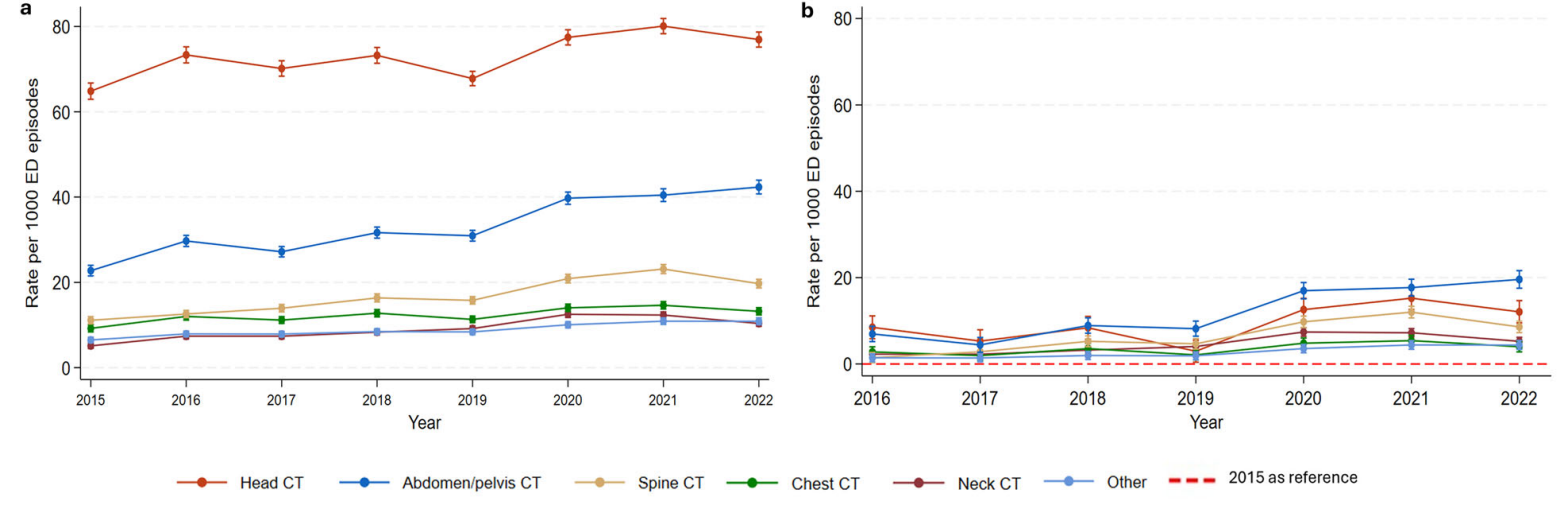
Trends in the rate of CT scanning by anatomical area. **a** The overall adjusted rate of CT scanning by anatomical area. **b** The difference in the rate for each anatomical area relative to its 2015 rate

Figure 2a displays the adjusted rates of CT scans per 1000 ED episodes by anatomical area. From 2015 to 2022, head CT showed the highest rates, followed by abdomen/pelvis scans, both of which were higher than rates for spine, chest, neck, and other. As illustrated in Fig. 2b, beginning in 2019, the absolute increase in abdomen/pelvis CT became more pronounced compared to head CT and all other types, reaching 19.58 [95% CI: 17.55, 21.62] in 2022, whereas head CT reached 12.08 [95% CI: 9.49, 14.67].

### Changes in admission

**Fig. 3:**
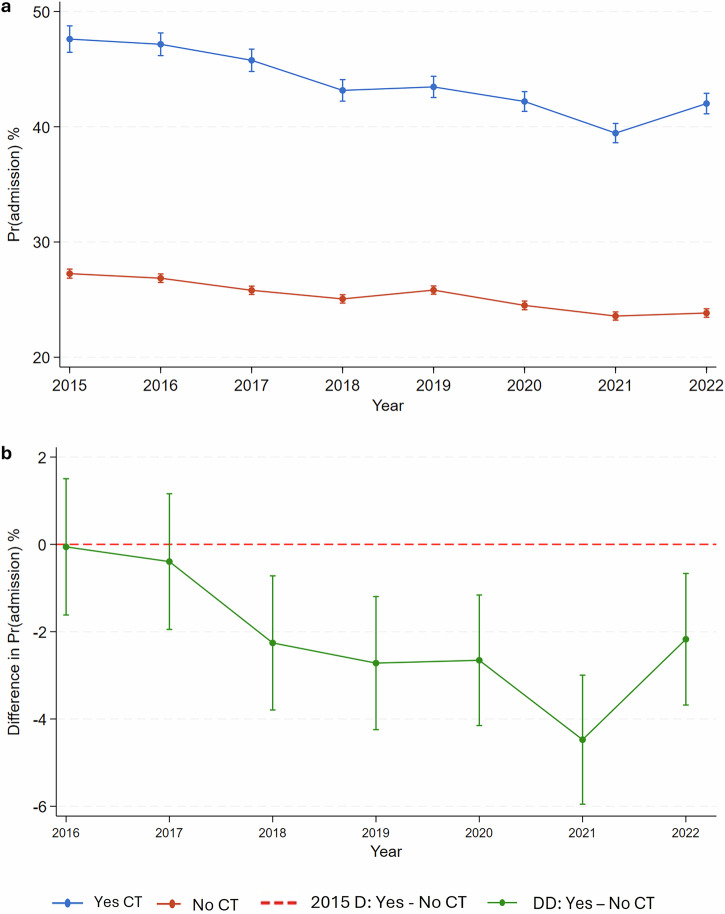
Trends in the probability of admission. **a** The overall adjusted probability of admission based on CT scanning status. **b** The difference in the probability of admission between with and without CT, compared to the 2015 difference between with and without. D, difference; DD, difference-in-difference

While the adjusted probability of hospital admission remained consistently higher for patients with CT, both groups experienced a decrease in admissions: from 47.60% [95% CI: 46.46, 48.75] in 2015 to 42.01% [95% CI: 41.12, 42.9] in 2022 for those with CT, and from 27.25% [95% CI: 26.86, 27.64] to 23.83% [95% CI: 23.47, 24.2] for those without CT (Fig. 3a). Compared to those without CT, the probability of admission for those with CT was 2.17% [95% CI: 3.68, 0.66] lower in 2022 than 2015 (Fig. 3b). Further details on the regression model for adjusted admission rates are shown in Table S3 (Supplementary Material).

## Discussion

In this study assessing trends in CT use in the ED from 2015 to 2022, we observed a 90% increase in the number of CT scans, significantly outpacing the 8% growth in ED episodes. The CT scans per 1000 ED episodes for this period increased by 76% (from 111 to 195) in observed data and by 47% (from 118 to 173) after adjustment. These findings suggest a continuation of previously reported trends in WA, where the adjusted rate increased from 56 in 2003 to 107 in 2015, as well as in other high-income countries across Europe, Asia, and North America [23, 31–34]. Furthermore, our findings align with recent trends from the United States, where CT scan rates in two EDs (Level I and Level III) increased substantially between 2014 and 2021 [35]. However, the rates reported in that study remained significantly higher than those in our study, rising from 250 to 340 per 1000 ED episodes (Level I) and from 270 to 480 (Level III) over the same period. Notably, this major difference may reflect the lack of adjustment for patient demographics and provider factors in that study (a limitation addressed in our analysis). Finally, the rise in CT use observed in the current study coincided with a significantly greater reduction in admission rate for those with CT compared to those without, consistent with previous literature [6, 36, 37].

The rate of CT use increased significantly, even after adjusting for variations in age, triage, Index of Relative Socioeconomic Advantage and Disadvantage, and other observed characteristics. This indicates that the residual increase is not simply attributable to changes in these factors but likely reflects changes in clinical practice, with a greater propensity for CT use.

Physician decision-making is likely influenced by interrelated factors. Efforts to manage patient flow and reduce overcrowding have been linked to increased CT use in EDs [38, 39]. Relevant to the current ED was the introduction of the Performance Management Policy in July 2019 [40]. This policy updated the monitoring framework for long-standing performance targets such as the Four-Hour Rule and the proportion of patients who left without being seen, by introducing remedial actions for non-compliance. These actions could range from external reviews to the removal of hospital representatives [40]. While aiming to make accurate decisions, physicians might have had to discharge patients more quickly and, as a result, may have been compelled to rely more on CT. This hypothesis is supported by both the reported relevance of CT in time-constrained scenarios and our findings, which show a significant and sustained increase in CT rates beginning in 2020, following the policy’s introduction [39]. However, as assessing the policy’s impact was beyond the scope of this study, further research is warranted.

Changes in clinical guidelines may have contributed to the increase in CT scans. During the study period, the local guidelines (Diagnostic Imaging Pathways) were actively promoted across clinical settings by the WA Department of Health until their discontinuation in April 2022 [41]. In mid-2019, some of these guidelines were updated, promoting the use of CT [41]. For instance, in the workup of suspected bowel obstruction, CT was recommended alongside the previously standard fluoroscopic examination. Coinciding with this particular update, our study observed a faster increase in abdomen/pelvis CT use from 2019 onward. Following the broader guideline changes, a sudden rise in overall CT use was observed from 2020. While guidelines are intended to support rather than dictate clinical judgment and may not always be strictly followed, it is plausible that adherence to the updated guidelines partly contributed to the rise in CT use.

Our study observed a sustained decrease in ED episodes during and following the onset of the COVID-19 pandemic in 2020, consistent with previous findings [42]. Pandemic-related restrictions led individuals to reduce their reliance on hospital care, opting instead for alternative healthcare options [43]. Many perceived their symptoms as mild and manageable, fostering a sustained trend of self-care that extended into 2021 and 2022 [43]. However, this shift was associated with the development of serious comorbidities [44]. Consequently, the increased use of CT scans observed in this study may be attributed to the initial lack of care-seeking for simpler conditions, which, due to inadequate self-management, later presented in a more complex and severe state.

The practice of defensive testing (ordering low-value tests to mitigate potential adverse outcomes) may also partly explain the increased use of CT [45]. Common drivers include fear of criticism from colleagues and medico-legal risks, with the latter accounting for an estimated 15–26% of CT orders [45, 46]. During the study period, complaints to the Health and Disability Services Complaints Office in WA regarding healthcare services, including EDs, rose from 1777 in 2015/16 to 2299 in 2021/22 [47]. Although most physicians may not have faced a complaint directly, this rising trend may have increased overall apprehension, potentially prompting many to order additional CT scans.

Technological advancements, such as iterative reconstruction, have led to significant reductions in CT radiation doses across all scan types in Australia between 2011 and 2017 [48]. Furthermore, Diagnostic Reference Levels, a benchmark set by the Australian Commission on Safety and Quality in Health Care to indicate whether radiation use for specific CT types is unusually high or low, were lowered in 2018 compared to 2012 [48]. Since the current radiology department is mandated to monitor and advised to optimize radiation doses when they exceed Diagnostic Reference Levels, it is possible that reductions in radiation doses occurred even without changes to the CT machines during the study period. This may have alleviated concerns about radiation safety, thereby normalizing the use of CT among physicians.

The faster decline in admissions among patients with CT suggests an increasing impact of CT on admissions since 2015. Supporting evidence shows that CT has influenced ED physicians’ decision-making, with 23.8% of patients discharged despite an initial plan for admission [49]. Consequently, current CT ordering practices may provide system-wide benefits, as using negative CT findings to prompt early discharge has been associated with reduced healthcare costs [50]. However, the use of observation units may have confounded our findings, as the decline in admissions could reflect transfers to these units rather than an actual reduction in hospital admissions. Additionally, consistent with previous hypotheses, the combination of increased CT use and reduced admissions among CT patients suggests expanded scanning of lower-risk patients who might not have been admitted otherwise, raising concerns about potential overuse [37].

Our findings provide the first examination of ED CT trends in Australia in light of recent efforts to reduce overuse, such as the Choosing Wisely campaign introduced in 2015 [23, 24]. The observation that trends in CT rates and admissions remain similar to pre-Choosing Wisely findings in Australia (and other countries) should prompt discussions on evaluating the campaign’s impact [6, 23, 36, 37]. Furthermore, the coincidence of increased CT use with a faster decline in admissions among patients undergoing CT may warrant quasi-experimental designs to explore whether policy changes contributed to this trend. Our study highlighted an increase in patients with risk factors associated with higher CT use, such as older age. However,  as we did not quantify the proportional contribution of these risk factors, further research is warranted.

### Limitations

A key limitation is the use of administrative data, which lacks clinical information, such as provisional diagnoses or reasons for ordering the scan. This prevented assessment of diagnostic yield and limited insight into the appropriateness of increased CT use. Furthermore, the data lacks information on external factors, such as changes in admission policies, which may have confounded the association between CT use and admission trends. However, we attempted to mitigate this using post-estimation predictive margins with contrast method, as outlined in previous research [29]. Another limitation of this study was the lack of linkage to other data sources, which restricted our ability to directly account for the severity component of clinical acuity or its proxy, the presence and number of comorbidities. However, we mitigated this by including age, Index of Relative Socioeconomic Advantage and Disadvantage, and Indigenous status as markers of underlying health status. Lastly, our study was conducted in a single ED and involved adults, which limits the generalizability of the findings to other settings and the broader population.

## Conclusion

This study observed a significant rise in CT scan rates from 2015 to 2022, with a marked upward trend beginning in 2020 and continuing onward. Over time, this trend coincided with a more pronounced decline in admission rates among patients with CT. Since this increase cannot be fully explained by changes in patient characteristics, it may result from practice changes driven by local guideline updates, the WA Performance Management Policy, and COVID-19. While current ordering practices may indicate cost savings from avoided hospitalizations, they could also suggest increased scanning of lower-risk patients and potential overuse. This study began with the implementation of Choosing Wisely in Australia in 2015, highlighting the need for a formal investigation into the campaign’s impact.

## Supplementary information

ELECTRONIC SUPPLEMENTARY MATERIAL

## Data Availability

The data analyzed in this study were provided directly by the institutions and are not publicly available due to the policy of the Human Research Ethics Committee. Access is restricted to the authors responsible for the analysis.

## Abbreviations

ED: Emergency department
WA: Western Australia
