# Supplementary material for: Trends in CT use in an emergency department in Western Australia: 2015–2022
Source: Insights Imaging. 2025 Jun 4;16:116. doi: 10.1186/s13244-025-01993-9 (PMC12137846; doi:10.1186/s13244-025-01993-9)
Supplement: Supplementary file 1 — ELECTRONIC SUPPLEMENTARY MATERIAL [file 13244_2025_1993_MOESM1_ESM.pdf]

Trends in CT use in an Emergency Department in Western Australia: 2015–2022

ELECTRONIC SUPPLEMENTARY MATERIAL

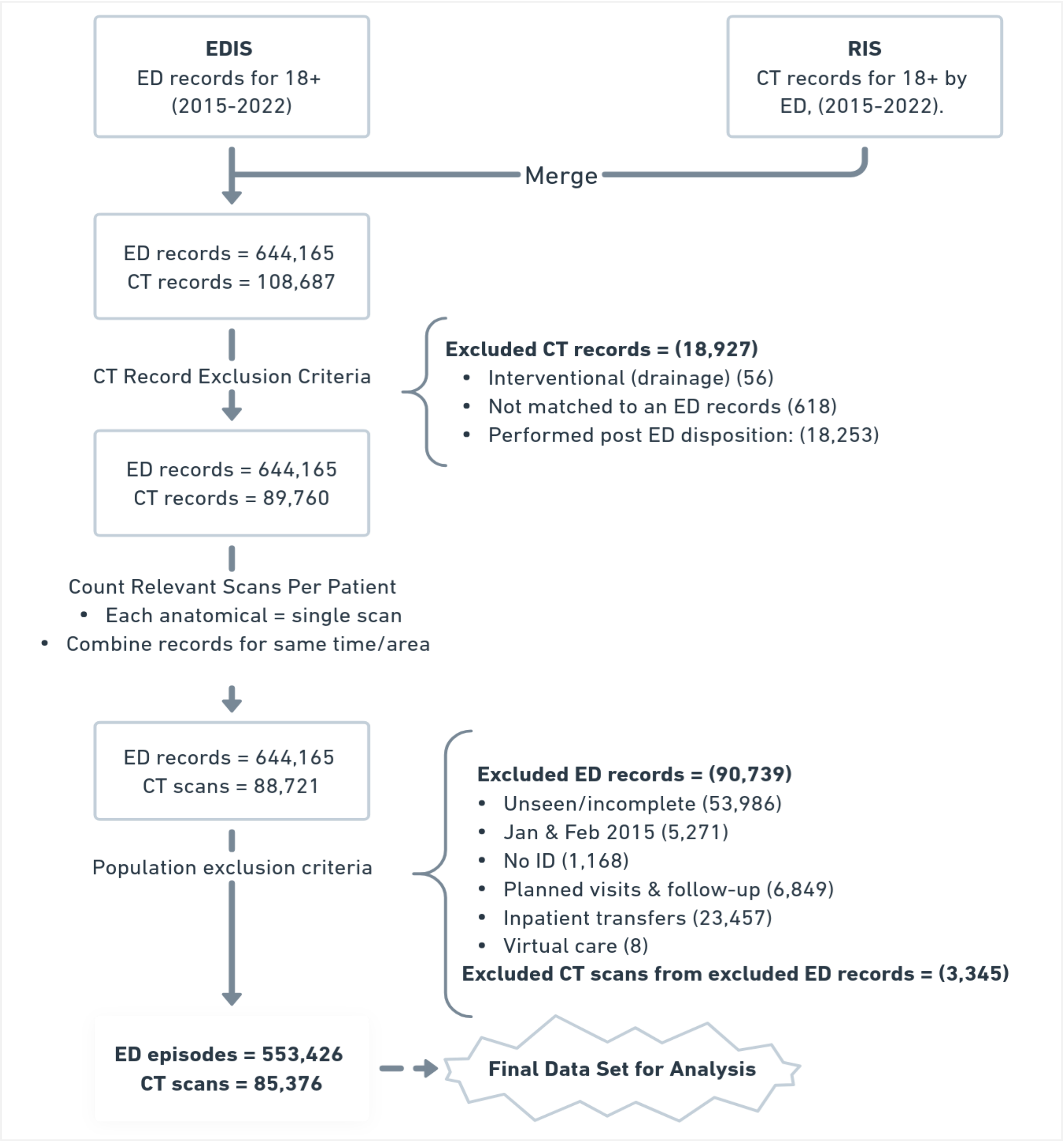

Fig.S1: Flowchart of CT and participant inclusion/exclusion. Abbreviations: EDIS = Emergency Department Information System, RIS = Radiology Information System, ED = Emergency Department.

Insights Imaging (2025) Almouqati F, Ha TN, Barnard S, et al.

Table S1: Numbers of CT scans and characteristics of the study population for each year during 2015-2022

	2015	2016	2017	2018	2019	2020	2021	2022	Total
N	58882 (10.6)	68571 (12.4)	71821 (13.0)	72107 (13.0)	76358 (13.8)	73096 (13.2)	69045 (12.5)	63546 (11.5)	553426 (100.0)
Overall CT	6541	9198	9384	10442	10880	12911	13592	12428	85376
Head	3500	4614	4705	5001	5162	5708	6135	5746	40571
Neck	256	442	469	571	691	966	988	816	5199
Chest	483	760	745	881	846	1046	1124	1004	6889
Spine	633	832	934	1115	1187	1513	1739	1428	9381
Abdomen/pelvis	1280	2000	1977	2280	2364	2949	2822	2737	18409
Other	389	550	554	594	630	729	784	697	4927
**Episode characteristics**									
**CT use status**									
No	53229 (90.4)	60811 (88.7)	63929 (89.0)	63563 (88.2)	67583 (88.5)	62910 (86.1)	58558 (84.8)	53864 (84.8)	484447 (87.5)
Yes	5653 (9.6)	7760 (11.3)	7892 (11.0)	8544 (11.8)	8775 (11.5)	10186 (13.9)	10487 (15.2)	9682 (15.2)	1168979 (12.5)
**Age group (years)**									
18-34	18793 (31.9)	20984 (30.6)	21627 (30.1)	21227 (29.4)	21986 (28.8)	20756 (28.4)	19370 (28.1)	17042 (26.8)	161785 (29.2)
35-54	16616 (28.2)	19083 (27.8)	19802 (27.6)	20035 (27.8)	21195 (27.8)	21149 (28.9)	19364 (28.0)	17369 (27.3)	154613 (27.9)
55-74	13048 (22.2)	15779 (23.0)	16890 (23.5)	17373 (24.1)	18322 (24.0)	17877 (24.5)	16319 (23.6)	15136 (23.8)	130744 (23.6)
75+	10425 (17.7)	12725 (18.6)	13502 (18.8)	13472 (18.7)	14855 (19.5)	13314 (18.2)	13992 (20.3)	13999 (22.0)	106284 (19.2)
**Median age (years)**[i]	47 (31-67)	48 (31-68)	48 (32-69)	49 (32-69)	49 (32-70)	49 (33-69)	50 (33-71)	51 (33-72)	49 (32-69)
**Sex**									
F	29787 (50.6)	35033 (51.1)	37235 (51.8)	37513 (52.0)	39541 (51.8)	37369 (51.1)	35563 (51.5)	32379 (51.0)	284420 (51.4)
M	29095 (49.4)	33538 (48.9)	34586 (48.2)	34594 (48.0)	36817 (48.2)	35727 (48.9)	33482 (48.5)	31167 (49.0)	269006 (48.6)
**Triage code**									
Semi/Non-urgent	25919 (44.0)	28699 (41.9)	28771 (40.1)	29008 (40.2)	30099 (39.4)	27365 (37.4)	23514 (34.1)	19780 (31.1)	213155 (38.5)
Urgent	21852 (37.1)	26167 (38.2)	28973 (40.3)	27699 (38.4)	29679 (38.9)	28658 (39.2)	26779 (38.8)	24937 (39.2)	214744 (38.8)
Resuscitation/Emergency	11111 (18.9)	13705 (20.0)	14077 (19.6)	15400 (21.4)	16580 (21.7)	17073 (23.4)	18752 (27.2)	18829 (29.6)	125527 (22.7)
**Symptom group**									
Other	11814 (20.1)	13539 (19.7)	14579 (20.3)	14471 (20.1)	15400 (20.2)	14527 (19.9)	13114 (19.0)	11668 (18.4)	109112 (19.7)
Cardiovascular	1415 (2.4)	1761 (2.6)	2170 (3.0)	2121 (2.9)	2206 (2.9)	2275 (3.1)	2197 (3.2)	2102 (3.3)	16247 (2.9)
Gastrointestinal	3301 (5.6)	3717 (5.4)	3918 (5.5)	3977 (5.5)	4101 (5.4)	3760 (5.1)	3471 (5.0)	3340 (5.3)	29585 (5.3)
Injury	11314 (19.2)	13134 (19.2)	12945 (18.0)	12841 (17.8)	13098 (17.2)	12444 (17.0)	12647 (18.3)	10967 (17.3)	99390 (18.0)
Neurological	5264 (8.9)	6212 (9.1)	6608 (9.2)	6807 (9.4)	7638 (10.0)	7495 (10.3)	7493 (10.9)	7334 (11.5)	54851 (9.9)

Pain	19196 (32.6)	22285 (32.5)	23457 (32.7)	23524 (32.6)	24559 (32.2)	23525 (32.2)	21858 (31.7)	19761 (31.1)	178165 (32.2)
Provisional diagnosis	1784 (3.0)	2269 (3.3)	2463 (3.4)	2621 (3.6)	3123 (4.1)	3077 (4.2)	2774 (4.0)	2476 (3.9)	20587 (3.7)
Respiratory	3710 (6.3)	4390 (6.4)	4412 (6.1)	4436 (6.2)	4895 (6.4)	4633 (6.3)	4304 (6.2)	4694 (7.4)	35474 (6.4)
Urology	1084 (1.8)	1264 (1.8)	1269 (1.8)	1309 (1.8)	1338 (1.8)	1360 (1.9)	1187 (1.7)	1204 (1.9)	10015 (1.8)
**Treating Clinician**									
Physician: Senior	17709 (30.1)	23666 (34.5)	28328 (39.4)	27622 (38.3)	25604 (33.5)	23409 (32.0)	19870 (28.8)	16101 (25.3)	182309 (32.9)
Doctor: Junior	34966 (59.4)	36903 (53.8)	36781 (51.2)	37004 (51.3)	43269 (56.7)	42679 (58.4)	41923 (60.7)	40016 (63.0)	313541 (56.7)
Other Clinicians	6207 (10.5)	8002 (11.7)	6712 (9.3)	7481 (10.4)	7485 (9.8)	7008 (9.6)	7252 (10.5)	7429 (11.7)	57576 (10.4)
**Referral Source**[a]									
Self/relative	50866 (86.4)	57691 (84.1)	59346 (82.6)	58279 (80.8)	61621 (80.7)	60422 (82.7)	57306 (83.0)	53596 (84.3)	459127 (83.0)
GP/OPD/Hospital	5397 (9.2)	7445 (10.9)	8408 (11.7)	8875 (12.3)	9058 (11.9)	7666 (10.5)	6960 (10.1)	5877 (9.2)	59686 (10.8)
Other	2499 (4.2)	3197 (4.7)	3421 (4.8)	3618 (5.0)	4640 (6.1)	4102 (5.6)	4232 (6.1)	3698 (5.8)	29407 (5.3)
**Arrival Means**									
Ambulance/police/flying	18614 (31.6)	21656 (31.6)	22484 (31.3)	22504 (31.2)	25565 (33.5)	23911 (32.7)	24791 (35.9)	24365 (38.3)	183890 (33.2)
Other	40268 (68.4)	46915 (68.4)	49337 (68.7)	49603 (68.8)	50793 (66.5)	49185 (67.3)	44254 (64.1)	39181 (61.7)	369536 (66.8)
**Presentation day**									
Weekday	41529 (70.5)	48111 (70.2)	50429 (70.2)	51056 (70.8)	54383 (71.2)	51989 (71.1)	49261 (71.3)	44727 (70.4)	391485 (70.7)
Weekend	17353 (29.5)	20460 (29.8)	21392 (29.8)	21051 (29.2)	21975 (28.8)	21107 (28.9)	19784 (28.7)	18819 (29.6)	161941 (29.3)
**Presentation shift**									
09:00-17:00	28749 (48.8)	34077 (49.7)	35282 (49.1)	35992 (49.9)	38042 (49.8)	37252 (51.0)	34107 (49.4)	32685 (51.4)	276186 (49.9)
17:01-23:59	19251 (32.7)	21760 (31.7)	23066 (32.1)	22860 (31.7)	24444 (32.0)	22585 (30.9)	22426 (32.5)	19338 (30.4)	175730 (31.8)
00:00-08:59	10882 (18.5)	12734 (18.6)	13473 (18.8)	13255 (18.4)	13872 (18.2)	13259 (18.1)	12512 (18.1)	11523 (18.1)	101510 (18.3)
**IRSAD**[a]									
Least disadvantaged	27846 (47.3)	32104 (46.8)	33458 (46.6)	33417 (46.3)	29816 (39.0)	28397 (38.8)	26592 (38.5)	23346 (36.7)	234976 (42.5)
Less	4845 (8.2)	5710 (8.3)	5923 (8.2)	5603 (7.8)	10575 (13.8)	10372 (14.2)	10120 (14.7)	9453 (14.9)	62601 (11.3)
Moderate	14416 (24.5)	16812 (24.5)	18004 (25.1)	18067 (25.1)	19828 (26.0)	18796 (25.7)	17822 (25.8)	16551 (26.0)	140296 (25.4)
Highly	6717 (11.4)	7881 (11.5)	8237 (11.5)	8637 (12.0)	8466 (11.1)	8214 (11.2)	7624 (11.0)	7362 (11.6)	63138 (11.4)
Most disadvantaged	3046 (5.2)	3691 (5.4)	3881 (5.4)	3947 (5.5)	5035 (6.6)	5137 (7.0)	5201 (7.5)	4969 (7.8)	34907 (6.3)
**Admission status**									
No	42829 (72.7)	49190 (71.7)	51773 (72.1)	52408 (72.7)	54695 (71.6)	52990 (72.5)	50105 (72.6)	45011 (70.8)	399001 (72.1)
Yes	16053 (27.3)	19381 (28.3)	20048 (27.9)	19699 (27.3)	21663 (28.4)	20106 (27.5)	18940 (27.4)	18535 (29.2)	154425 (27.9)

Note: Unless otherwise noted, data are reported as n (%). Indigenous status is not reported due to ethics requirements; adjustments applied. **Abbreviations:** GP = general practitioner; OPD = outpatient department; IRSAD = Index of Relative Socioeconomic Advantage and Disadvantage.

[a]Totals do not sum to 100% due to missing data.

[i] Reported as median age (IQR: 25th-75th)

Table S2: Multivariable negative binomial regression results for trend in (overall) CT use

	IRR	95%CI	PValue
Year			
2015	1		
2016	1.2	(1.16, 1.24)	<.001
2017	1.16	(1.12, 1.19)	<.001
2018	1.27	(1.23, 1.31)	<.001
2019	1.21	(1.17, 1.25)	<.001
2020	1.48	(1.43, 1.53)	<.001
2021	1.53	(1.48, 1.58)	<.001
2022	1.46	(1.41, 1.5)	<.001
**Age group (years)**			
18-34	1		
35-54	1.67	(1.62, 1.72)	<.001
55-74	2.15	(2.1, 2.21)	<.001
75+	2.25	(2.19, 2.32)	<.001
**Sex**			
F	1		
M	1.15	(1.13, 1.17)	<.001
**Triage code**			
Semi-Urgent/Non-Urgent	1		
Urgent	2.17	(2.12, 2.21)	<.001
Resuscitation/Emergency	2.76	(2.7, 2.83)	<.001
**Symptom group**			
Other	1		
Cardiovascular	0.85	(0.79, 0.91)	<.001
Gastrointestinal	1.39	(1.32, 1.45)	<.001
Injury	4.69	(4.53, 4.86)	<.001
Neurological	5.37	(5.18, 5.55)	<.001
Pain	1.93	(1.86, 2)	<.001
Provisional diagnosis	1.18	(1.11, 1.25)	<.001
Respiratory	0.76	(0.72, 0.8)	<.001
Urology	1.06	(0.98, 1.15)	0.133
**Treating Clinician**			
Physician: Senior	1		
Doctor: Junior	1.04	(1.02, 1.05)	<.001
Other Clinicians	0.19	(0.17, 0.2)	<.001
**Referral Source**			
Self/relative	1		
GP/OPD/Hospital	1.01	(0.99, 1.04)	0.328

Other	1.16	(1.13, 1.19)	<.001
**Arrival Means**			
Ambulance/police/flying	1		
Other	0.68	(0.67, 0.69)	<.001
**Presentation day**			
Weekday	1		
Weekend	1.01	(0.99, 1.02)	0.366
**Presentation shift**			
09:00-17:00	1		
17:01-23:59	0.97	(0.95, 0.98)	<.001
00:00-08:59	1.06	(1.04, 1.08)	<.001
**IRSAD**			
Least disadvantaged	1		
Less disadvantaged	1.04	(1.01, 1.07)	0.003
Moderate disadvantaged	1.02	(1, 1.04)	0.025
Highly disadvantaged	1.04	(1.02, 1.07)	0.001
Most disadvantaged	1.11	(1.07, 1.14)	<.001

Abbreviations: IRR = incident rate ratio, GP = general practitioner; OPD = outpatient department; IRSAD = Index of Relative Socioeconomic Advantage and Disadvantage.

Table S3: Multivariable logistic regression results assessing changes in admission by CT scan status

**Year**	OR	95%CI	PValue
2015	1		
2016	0.98	(0.95, 1.01)	0.124
2017	0.91	(0.88, 0.94)	<.001
2018	0.87	(0.84, 0.9)	<.001
2019	0.91	(0.88, 0.94)	<.001
2020	0.84	(0.81, 0.87)	<.001
2021	0.79	(0.76, 0.81)	<.001
2022	0.8	(0.77, 0.83)	<.001
**CT use status**			
No	1		
Yes	3.06	(2.87, 3.26)	<.001
**Year#CT**			
2015#No	1		
2015#Yes	1		
2016#No	1		
2016#Yes	1	(0.92, 1.09)	0.968
2017#No	1		
2017#Yes	1	(0.92, 1.08)	0.952
2018#No	1		
2018#Yes	0.92	(0.84, 0.99)	0.032
2019#No	1		
2019#Yes	0.88	(0.82, 0.96)	0.003
2020#No	1		
2020#Yes	0.9	(0.83, 0.98)	0.012
2021#No	1		
2021#Yes	0.83	(0.77, 0.9)	<.001
2022#No	1		
2022#Yes	0.94	(0.86, 1.01)	0.104
**Age groups (year)**			
18-34	1		
35-54	1.25	(1.22, 1.29)	<.001
55-74	2.18	(2.12, 2.24)	<.001
75+	3.71	(3.6, 3.82)	<.001
**Sex**			
F	1		
M	1.15	(1.13, 1.17)	<.001
**Triage group**			
Semi-Urgent/Non-Urgent	1		
Urgent	2.05	(2.01, 2.09)	<.001

Resuscitation/Emergency	3.01	(2.94, 3.08)	<.001
**Symptom group**			
Other	1		
Cardiovascular	0.7	(0.67, 0.73)	<.001
Gastrointestinal	1.62	(1.57, 1.68)	<.001
Injury	0.39	(0.37, 0.4)	<.001
Neurological	0.73	(0.71, 0.75)	<.001
Pain	0.57	(0.56, 0.59)	<.001
Provisional diagnosis	1.66	(1.6, 1.72)	<.001
Respiratory	1.94	(1.88, 2.01)	<.001
Urology	0.75	(0.71, 0.79)	<.001
**Treating Clinician**			
Physician: Senior	1		
Doctor: Junior	0.61	(0.6, 0.62)	<.001
Other Clinicians	0.41	(0.4, 0.43)	<.001
**Referral Source**			
Self/relative	1		
GP/OPD/Hospital	1.87	(1.83, 1.91)	<.001
Other	0.91	(0.88, 0.94)	<.001
**Arrival Means**			
Ambulance/police/flying	1		
Other	0.59	(0.57, 0.6)	<.001
**Presentation day**			
Weekday	1		
Weekend	0.93	(0.91, 0.94)	<.001
**Presentation shift**			
09:00-17:00	1		
17:01-23:59	0.91	(0.89, 0.92)	<.001
00:00-08:59	0.89	(0.87, 0.9)	<.001
**IRSAD**			
Least disadvantaged	1		
Less disadvantaged	1.17	(1.14, 1.21)	<.001
Moderate disadvantaged	1.16	(1.13, 1.19)	<.001
Highly disadvantaged	1.5	(1.46, 1.54)	<.001
Most disadvantaged	1.81	(1.75, 1.87)	<.001

Abbreviations: OR = odds ratio, GP = general practitioner; OPD = outpatient department; IRSAD = Index of Relative Socioeconomic Advantage and Disadvantage.
